# Utilizing Hyperbaric Oxygen Therapy to Improve Cognitive Function in Patients With Alzheimer’s Disease by Activating Autophagy-Related Signaling Pathways

**DOI:** 10.33549/physiolres.935447

**Published:** 2025-02-01

**Authors:** Binbin LI, Haizhen LI, Houhuang CHEN, Yanfang SUI, Ji ZENG, Xiafei LIN, Qianqian FAN, Zhenhua SONG

**Affiliations:** 1 Department of Rehabilitation Medicine, The Affiliated Haikou Hospital of Xiangya Medical College, Central South University, Haikou, Hainan, China; 2 Department of Radiology, Orthopedics and Diabetes Hospital in Haikou, Shanghai Sixth Peoples Hospital, Haikou, Hainan, China

**Keywords:** Alzheimer’s disease, Autophagy, Hyperbaric oxygen, Morris water maze, PCR

## Abstract

To investigate the impact of hyperbaric oxygen therapy (HBOT) on the cognitive function of mice with Alzheimer’s disease (AD), while also identifying the cellular pathways associated with autophagy involved in the treatment. Twenty-four APP/PSl double transgenic mice were randomly assigned to either Group A or Group B, while another 24 C57 mice were randomly allocated to Group C or Group D. HBOT was administered to mice in Group B and Group D, and the Morris water maze test was used to assess changes in mice behavior. Histological examination using hematoxylin and eosin staining was conducted to observe pathological alterations in the hippocampus of the mice brain tissue. Polymerase chain reaction (PCR) was employed to analyze autophagy-related gene pathways in the hippocampus of the mice. Following HBOT, mice in Group B exhibited a significant reduction in escape latency and a notable increase in residence time within the target quadrant compared with Group A (P<0.05), as well as Group C and Group D (P<0.01). The hippocampal neurons in Group A and Group B mice exhibited disorganized arrangements, characterized by pyknosis and margination. Conversely, neurons in Group C displayed orderly arrangements, retaining intact structures with round nuclei demonstrating clear nuclear staining and normal morphology. The cellular morphology of mice in Group D remained unaffected. PCR analysis revealed no notable disparity in autophagy-related gene expression between Group A and Group C. However, the expression levels of five genes including Tgfb1, Mapk14, Bid, Atg7, and Akt1, were significantly elevated in Group B compared to Group A. HBOT has the potential to improve the cognitive function in mice modeled with AD. This improvement of cognitive function appears to be mediated by the up-regulation of autophagy-related genes, specifically Tgfb1, Mapk14, Bid, Atg7, and Akt1. These results indicate that HBOT may offer a therapeutic strategy for treating AD by enhancing autophagy mechanisms.

## Introduction

Alzheimer’s disease (AD) is a prevalent neurodegenerative disorder of the brain observed predominantly among the elderly, characterized by gradual cognitive decline and memory impairment [1]. The pathogenesis of AD remains elusive. Presently, its pathological manifestations primarily involve the abnormal accumulation of β-amyloid leading to the formation of senile plaques, hyperphosphorylation of Tau protein resulting in the formation of neurofibrillary tangles (NFTs), and loss of cholinergic neurons [2]. The progressive advancement of AD significantly impacts the quality of life of affected individuals. However, there is currently no established therapeutic regimen capable of reversing disease progression or improve treatment efficacy [3].

Hyperbaric oxygen therapy (HBOT) directly impacts the cerebral internal environment by augmenting oxygen levels in the bloodstream, mitigating hypoxic conditions within the body, and accelerating the restoration of damaged neural tissues, thereby eliciting notable improvements in central nervous system function [4]. Research indicates that hypoxia plays a role in the pathogenesis of AD, and that interventions targeting hypoxia can potentially delay or alleviate the onset of neurodegenerative disorders [5]. Both clinical observations and experimental studies have demonstrated the capacity of hyperbaric oxygen (HBO) to improve the clinical symptoms and pathophysiological damage in AD model mice [6]. However, the precise mechanism underlying this therapeutic effect remains unclear.

Autophagy, acellular process involving self-phagocytosis followed by lysosomal degradation is essential for maintaining cellular homeostasis. HBOT facilitates the progression of autophagy by increasing autophagosome formation, promoting the fusion of lysosomes with autophagosomes, and protecting lysosomal integrity, thereby exerting neuroprotective effects. It is currently hypothesized that autophagy may contribute to the therapeutic efficacy of HBOT in AD.

In this study, experiments were conducted on animals, and an AD animal model was established to examine the impact of HBOT on cognitive function and associated autophagy pathways in AD model mice. A new theoretical basis for clinical hyperbaric oxygen therapy for AD.

## Materials and Methods

### Materials

The experimental cohort consisted of 5-month-old APP/PS1 double transgenic model mice (sourced from Beijing Viewsolid Biotechnology Co., Ltd.) and C57BL/6 mice (sourced from Zhejiang Weitong Lihua Co., Ltd.), with 24 mice of each strain weighing between 25 g to 30 g. There were a total of 48 experimental mice, with 12 mice per group. PCR was used to analyze gene expression across 84 samples, corresponding to each mouse. These mice were housed in a specific pathogen-free environment, with 4 mice per cage. They were provided *ad libitum* access to food and water and maintained at a room temperature ranging from 20–28 °C, with a humidity level of 40–60 %. The mice were subjected to a natural day-night cycle, with 12 h of light per day. Bedding, food, and drinking water, which had undergone rigorous disinfection with ultraviolet rays, were replaced weekly. The animal experimental protocols were reviewed and approved by the Ethics Committee of Haikou People’s Hospital (NO. 2021-108). All surgical procedures were performed under anesthesia to minimize pain, suffering, and mortality among experimental animals.

#### Key reagents and instruments

Key reagents and instruments utilized in the study included an animal HBO chamber sourced from Shanghai Tawang Technology Co., Ltd., a digital camera, hematoxylin and eosin (HE) staining reagents procured from Sigma, USA, an autophagy polymerase chain reaction (PCR) chip obtained from Shanghai Biochip Co., Ltd., qPCR reagents from Vazyme, a Morris water maze acquired from Anhui Zhenghua Biologic Apparatus Facilities Co., Ltd.), and a fluorescence quantitative PCR instrument from Thermo, among others.

### Methods

#### Grouping of experimental animals

AD mice were allocated to Group A and Group B, while C57BL/6 mice were assigned to Group C and Group D, each group comprising 12 mice. Upon grouping, individual mice were appropriately labeled for identification purposes. Mice in Group A and Group C were maintained under standard feeding conditions without any intervention, whereas mice in Group B and Group D received HBOT. Subsequent to the intervention period, samples were collected from each group for the assessment of relevant parameters.

#### Morris water maze

Morris water maze behavior observation typically consists of two main tasks: spatial probe and place navigation. Prior to commencing the experiment, spatial navigation was conducted on the mice across all four groups over a period of three days, with four sessions per day at fixed intervals. The Morris water maze, segmented into four quadrants (I, II, III, IV), served as the experimental apparatus. Initially, the mice were introduced into the pool without the presence of a platform to allow them to acclimatize to the maze environment during a 2-minute free swim session. During the training phase, a platform was placed within quadrant IV, and mice were placed into the pool, facing the pool wall, at one of the four designated starting points along the perimeter. The escape latency, denoting the time taken by the mice to locate the platform, along with their swimming trajectory, was recorded using a free video recording system. During the four training sessions, mice were released into the water from four distinct starting points corresponding to different quadrants of the maze.

During the Morris water maze experiment, if the mice successfully located the platform or failed to do so within the allocated time of 120 s (with the escape latency recorded as 120 s), the researcher would guide the mice to the platform and allow them to rest on it for 15 s before proceeding to the next experiment. The average escape latency across the 4 training sessions was calculated and recorded as the mice’s learning performance for that particular day.

During the spatial probe test conducted on the 4^th^ day, the original platform was removed from the Morris water maze, and the mice were introduced into the water from a consistent entry point. All mice were subjected to the same entry point for consistency. The proportion of time spent by each mouse crossing the quadrant where the original platform was located, to the total duration of their activity was recorded.

Following HBOT, the Morris water maze test was conducted after a 2-day period of normal feeding. This assessment encompassed both spatial probe and place navigation tasks, utilizing the same procedures as previously described.

#### HBO intervention

Following the initial Morris water maze test, mice in Group B and Group D were subjected to HBOT within the HBO chamber. The therapy regimen comprised daily sessions of 100 % pure oxygen at 2 atmospheric absolute (ATA) for a duration of 1 h each [7–11]. Both, pressurization and decompression processes were conducted slowly over a period of 10 min each. This therapeutic protocol consisted of two cycles, each spanning 10 days. Meanwhile, mice in Group A and Group C were also placed in the HBO chamber; however, they did not receive any treatment. Throughout the intervention period, the general condition of the mice was closely monitored and documented.

#### HE staining to observe hippocampal neuronal morphology

Upon completion of the Morris water maze test, mice were euthanized *via* cervical dislocation, and their brain tissues were extracted and divided into left and right cerebral hemispheres. The hippocampal tissue from one hemisphere was isolated and preserved in a refrigerator at −80 °C for subsequent analysis. The other half of the brain tissue was fixed in paraformaldehyde for 4 h, followed by dehydration using ethanol and embedding in paraffin to facilitate the production of thin sections measuring 5 μm in thickness. The sections were dewaxed, dehydrated, and subjected to staining using an HE staining kit. The morphology of hippocampal neurons was then assessed under a light microscope.

#### Autophagy PCR microarray analysis

Hippocampal tissues were carefully chosen, and total RNA was extracted and reverse transcribed. The gene expression profiles of autophagy-related molecules were then analyzed utilizing an Autophagy PCR Array kit. Comparative assessments of gene expression patterns among the four experimental groups of mice were conducted. Subsequently, through bioinformatics analysis, candidate key autophagy genes and signaling pathways influenced by HBO were identified and screened.

### Statistical analysis

Statistical analysis was performed using SPSS 26.0 software for data processing. Measurement data are presented as mean ± standard deviation (X±S), and comparison between groups were conducted using the *t*-test. The significance level was set at α=0.05, with P<0.05 indicating statistical significance.

## Results

### General observations

Throughout the duration of the experiment, mice in all experimental groups maintained normal dietary intake, and displayed smooth and shiny fur. Notably, there were no occurrences of mortality among the mice within any of the experimental groups during the course of the experiment.

### Effect of HBO on the cognitive function of mice in each group

#### Comparison of Morris water maze results in mice across the 4 groups

Prior to the experiment, all groups of mice group exhibited difficulty in locating the submerged platform during the place navigation test. After 4 days of training, the escape latency of mice in each group was gradually shortened. Mice in Group A and Group B continued to display significantly longer escape latency compared to those in Group C and Group D (P<0.01). No significant difference in escape latency was observed between Group A and Group B, nor between Group C and Group D. Following HBOT, Group B mice showed a significant reduction in escape latency and an increase in target quadrant residence time compared to Group A (P<0.05) and Group C and Group D (P<0.01). No significant difference in escape latency or target quadrant resistance time was found between Group C and Group D (Table 1).

#### HE staining

**Fig. 1:**
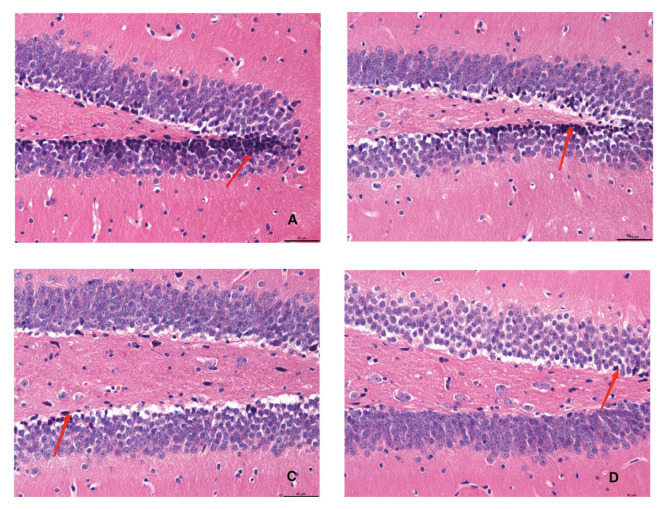
Mouse hippocampal neuron morphology (bar=50 μm), arrow indicates neurofibrillary tangles.

Figure 1 illustrates the cellular morphology of hippocampal neurons in the different experimental groups. In Group A and Group B, hippocampal neurons exhibited a disorganized arrangement, characterized by pyknosis and margination. Conversely, neurons in mice from Group C displayed an orderly arrangement, featuring intact structures and round nuclei with clear nuclear staining, indicative of normal morphology. The cellular morphology of mice in Group D appeared normal.

#### PCR autophagy chip test results

**Fig. 2:**
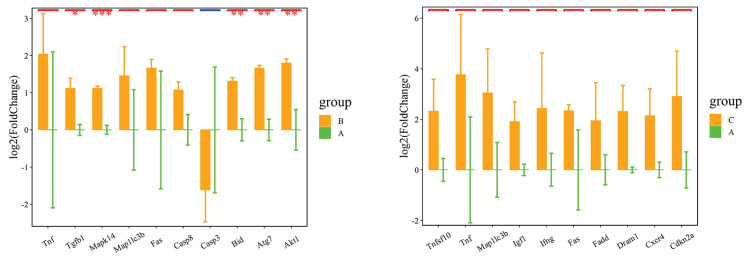
Red color indicates up-regulated genes, blue color indicates down-regulated genes, and gray indicates genes that are not significantly different, * indicates P<0.05, ** indicates P<0.01, *** indicates P<0.001.

Figure 2 depicts the results of autophagy PCR chip analysis conducted on total RNA extracted from the hippocampus of experimental mice. Compared to Group A, Group B exhibited significant up-regulation in the expression levels of five genes, namely Tgfb1, Mapk14, Bid, Atg7, and Akt1. No significant difference in the expression of autophagy-related genes was observed between Group A and Group C.

## Discussion

AD is the most prevalent prevalent neurodegenerative disorder globally [12]. The primary therapeutic approaches for AD include the use of cholinesterase inhibitors, calcium-modulated phosphatase and glutamate receptor antagonists [13]. However, due to the constraints of efficacy and adverse effects, there has been a growing interest in non-pharmacological interventions for AD.

In recent years, hyperbaric oxygen has been used to treat neurological and neurodegenerative diseases, as well as to improve cognitive function and cerebral metabolism in the presence of mild cognitive dysfunction [14]. Yang *et al*. [15] found that long-term HBOT reduced cognitive impairment in AD mice by treating them with HBOT interventions for 3 consecutive months, and was effective in decreasing the deposition of

Aβ plaques in AD mice, hyperphosphorylated tau protein aggregation and the progression of neuronal and synaptic degeneration in AD mice. Our group evaluated the learning and memory ability of AD model mice by HBO intervention and water maze experiment. The results showed that the avoidance latency of the HBO-intervened AD model mice was shorter than that of the non-intervened group, and the residence time in the target quadrant was longer, indicating that HBO could improve the cognitive function of AD mice. Meanwhile, the HE staining of the hippocampal tissues showed that the intranuclear solidification was reduced in the HBO-intervened AD mice compared with the other AD mice, suggesting that HBO could reduce the hippocampal damage and improve the cognitive function of AD mice.

Based on the cellular autophagy hypothesis in AD research, which suggests that autophagy participates in β-amyloid degradation and that impaired autophagic microfunction during AD pathology leads to a large accumulation of Aβ in neurons. This results in neuronal damage and symptoms associated with Alzheimer’s disease [16]. Our previous study found that sleep deprivation could promote the aggravation of cognitive dysfunction in APP/PS1 double transgenic mice, leading to morphological alterations of hippocampal neuronal cells and causing an increase in the expression of senile plaques formed by Aβ42 aggregates in the hippocampus and temporal lobe cortex of mice; and sleep deprivation induced the enhancement of autophagic activity in hippocampal tissues of mice, which may be a mechanism that mediates the onset and progression of AD. Chuanfen *et al*. [17] found that key proteins of autophagy were altered after hyperbaric oxygen treatment by establishing a rat ischemia-reperfusion model, confirming that hyperbaric oxygen treatment had an effect on autophagic response, showing the role of autophagy in ischemic stroke. Our group extracted total RNA from hippocampal tissues after hyperbaric oxygen treatment of AD model mice for Autophagy PCR microarray detection, a total of 84 groups were monitored, and it was found that the difference of autophagy-related genes was not statistically significant in Group A compared with Group C, indicating that autophagy genes were not expressed in AD mice, and that the signals of five genes, including Tgfb1, Mapk14, Bid, Atg7, and Akt1, were up-regulated in the hippocampal tissue-extracted total RNA in Group B compared with that of Group A, and the difference was statistically significant. It indicates that autophagy genes are induced to be upregulated by external intervention and involved in AD treatment. Our next step will be to further study the related mechanism of hyperbaric oxygen therapy for AD on the related autophagy gene pathway. For example, the mechanism study of hyperbaric oxygen therapy for cognitive dysfunction based on near-infrared brain functional imaging.

In summary, hyperbaric oxygen therapy significantly improves cognitive function in Alzheimer’s disease AD mice, and genes such as Tgfb1, Mapk14, Bid, Atg7, and Akt1 may play an important role in autophagy process, which provides a theoretical basis for the clinical application of HBO in the treatment of AD.

## Conclusions

HBOT emerges as a promising intervention for enhancing the cognitive function of AD mice. Furthermore, we identified several genes, namely Tgfb1, Mapk14, Bid, Atg7, and Akt1, which may be pivotal in the autophagic process associated with HBOT treatment. Our study provides a theoretical foundation for the clinical application of HBOT in AD treatment.

## Figures and Tables

**Table 1:** Results of Morris water maze experiments.

Items and groups		A	B	C	D
*Escape latency*	*Before intervention*	36.18±20.05	22.86±15.26	11.02±3.88**,#	10.69±3.29**,##
	*After intervention*	31.51±3.93	26.84±4.19*	8.42±2.43**,##	10.88±6.40**,##

*Target quadrant residence time*	*Before intervention*	29.41±0.97	29.89±1.61	34.27±0.17**,##	34.25±0.19**,##
	*After intervention*	29.39±0.43	30.62±1.35*	34.23±0.46**,##	34.10±0.29**,##

* indicates P<0.05 compared to Group A,

** indicates P<0.01 compared to Group A,

# indicates P<0.05 compared to Group B,

## indicates P<0.01 compared to Group B.

## Abbreviations

Aβ: Amyloid
AD: Alzheimer’s disease
ATA: Atmosphere absolute
HBO: Hyperbaric oxygen
HBOT: Hyperbaric oxygen therapy
NFTs: Neurofibrillary tangles
PCR: Polymerase Chain Reaction.
